# Adoption of Stroke Rehabilitation Technologies by the User Community: Qualitative Study

**DOI:** 10.2196/rehab.9219

**Published:** 2018-08-17

**Authors:** Andrew Kerr, Mark Smith, Lynn Reid, Lynne Baillie

**Affiliations:** ^1^ Centre of Excellence in Rehabilitation Research Department of Biomedical Engineering University of Strathclyde Glasgow United Kingdom; ^2^ Stroke Rehabilitation Unit Physiotherapy Department NHS Lothian Edinburgh United Kingdom; ^3^ Education Programmes Chest Heart & Stroke Scotland Edinburgh United Kingdom; ^4^ Interactive and Trustworthy Technologies Department of Mathematical and Computer Sciences Heriot-Watt University Edinburgh United Kingdom

**Keywords:** stroke, rehabilitation, technology, priorities

## Abstract

**Background:**

Using technology in stroke rehabilitation is attractive. Devices such as robots or smartphones can help deliver evidence-based levels of practice intensity and automated feedback without additional labor costs. Currently, however, few technologies have been adopted into everyday rehabilitation.

**Objective:**

This project aimed to identify stakeholder (therapists, patients, and caregivers) priorities for stroke rehabilitation technologies and to generate user-centered solutions for enhancing everyday adoption.

**Methods:**

We invited stakeholders (n=60), comprising stroke survivors (20/60, 33%), therapists (20/60, 33%), caregivers, and technology developers (including researchers; 20/60, 33%), to attend 2 facilitated workshops. Workshop 1 was preceded by a national survey of stroke survivors and therapists (n=177) to generate an initial list of priorities. The subsequent workshop focused on identifying practical solutions to enhance adoption.

**Results:**

A total of 25 priorities were generated from the survey; these were reduced to 10 nonranked priorities through discussion, consensus activities, and voting at Workshop 1: access to technologies, ease of use, awareness of available technologies, technologies focused on function, supports self-management, user training, evidence of effectiveness, value for money, knowledgeable staff, and performance feedback. The second workshop provided recommendations for improving the adoption of technologies in stroke rehabilitation: an annual exhibition of commercially available and developing technologies, an online consumer-rating website of available technologies, and a user network to inspire and test new technologies.

**Conclusions:**

The key outcomes from this series of stakeholder workshops provides a starting point for an integrated approach to promoting greater adoption of technologies in stroke rehabilitation. Bringing technology developers and users together to shape future and evaluate current technologies is critical to achieving evidence-based stroke rehabilitation.

## Introduction

### Background

Stroke has been a priority for the National Health Service (NHS) in Scotland for the last 15 years. In that time, there has been a 21% decrease in incidence and a 41% improvement in survival rates [1]. These figures represent an enormous success for public health and acute care but have created a new challenge: to provide rehabilitation and care to the increasing number of survivors, currently estimated at 117,500 in Scotland [2]. This challenge is not confined to Scotland; worldwide, an estimated 15 million people suffer from stroke every year, a third of whom are estimated to be left with persistent disability [3].

There is good evidence that rehabilitation can improve recovery from stroke [4]. The recovery of specific functions such as walking and upper limb activities are improved through repetitive, task-specific practice with performance feedback [5], all delivered, typically, by rehabilitation professionals. While rationing access to such a resource is understandable in the context of health budget constraints, this is likely to limit the recovery of some individuals.

In response to this need, technology has been used to increase rehabilitation practice intensity [6,7], enhance health professionals’ efficiency [8], and provide objective feedback on progress [9]. Technology can also support independent practice, which is critical to achieving the levels of intensity associated with improved outcomes [8]. Technologies are developing rapidly, and global advances in digital healthcare mean that a greater reliance on technology is inevitable. In Scotland, this is compounded by the drive to reduce the length of hospital stay [10], which will, by necessity, require greater integration of care in the community and promotion of self-management [11]. Technologies designed to promote patient-centered functional recovery after stroke can play a critical role, particularly in those aspects prioritized by patients and healthcare professionals (eg, mobility, speech, cognition, and confidence) [12]. Currently, few of these technologies are being embedded into everyday practice. This may relate to technology developers focusing on impairment, and not on the functional needs of the individual [13], as well as a general lack of collaboration across the stakeholders, (ie, users, technology developers including researchers, and policymakers) [14].

A perceived mismatch between research and patient priorities for life after stroke motivated a Priority Setting Partnership (PSP) that produced a list of agreed priorities for future research [12] that has been widely adopted by the research community. This was considered a sensible first step to resolving the poor adoption of technologies in stroke rehabilitation.

Our aim was to identify stakeholder priorities for stroke rehabilitation technologies using an adapted version of the James Lind Alliance approach to priority setting [15] and then use these priorities to generate user-centered solutions to enhance the everyday adoption of technologies by users, therapists, patients, and caregivers.

### Objectives

The objectives were (1) to gather stakeholder priorities for the development of technologies in stroke rehabilitation, (2) to produce a top 10 list of priorities through a process of consensus across stakeholders, and (3) to generate new ideas from stakeholders on ways to improve the adoption of technology in stroke rehabilitation.

## Methods

To achieve our aim, we planned a consensus-building process that emulated the James Lind PSP [15]. This consisted of a national survey of stakeholders to gather a long list of priorities followed by two one-day workshops inviting local, national, and international stakeholders. While Workshop 1 followed the James Lind process to reach consensus on the top 10 priorities, Workshop 2 aimed to generate new ideas using the top 10 list as a framework. An organizing committee consisting of 2 stroke survivors, 2 NHS therapists, 2 researchers, and 1 representative from the third sector, the charitable organization Chest Heart and Stroke Scotland (CHSS), agreed with the overall aim of the project, the design of the survey, and the structure of the two workshops. The study was ethically approved by the University Ethics Committee of University of Strathclyde (UEC16/02).

## Results

### Stakeholder Survey

Surveys were sent to stroke survivors, caregivers, and rehabilitation professionals working in stroke to generate a long list of priorities from the broad community. These surveys were distributed electronically and manually through professional (Scottish Allied Health Professions Forum) and patient support networks (CHSS) to reach as broad a population as possible. As the survey was designed with the single purpose of generating a long list of priorities, only 6 questions were posed. These included background information on the use of rehabilitation technologies and a request to state their perceived priorities for stroke rehabilitation technologies. A copy of the survey can be found in Multimedia Appendix 1. The response to the request for priorities provided 137 individual priorities. These were checked for duplication, and a list of priorities was assembled and ranked by popularity. To be included on the final list, a priority had to be stated by at least two individuals. In this way, a list of 25 ranked priorities was produced from the survey.

### Stakeholder Workshop 1: Consensus Agreement

Workshop 1 was located at a neutral (ie, not a hospital or university), city center venue with good public transport links and disabled access. The workshop lasted 7 hours with breaks for lunch and refreshments. Sixty delegates representing the three stakeholder groups (users, technology developers including researchers, and policymakers) attended the workshop. Delegates were recruited through general invitations sent out to members of the Scottish Allied Health Professions Forum (therapists), CHSS patient networks (patients and caregivers), and individuals known to the committee as being active and experienced in this area (policymakers, researchers, and technology developers). The final delegate list was agreed upon by the organizing committee to ensure an even proportion from each group. Delegates were placed at 7 tables so that each table had at least 2 individuals from each of the stakeholder groups and a facilitator. Facilitators experienced in working with stroke survivors and therapists were supplied by CHSS.

The workshop included presentations of different models of rehabilitation provision including community therapy delivered according to the current NHS model, private rehabilitation delivered in the patient’s home, and a third sector (charitable organization) service based around a gym and activity center, which made use of technologies such as virtual reality. There were also short demonstrations of rehabilitation technologies designed for mobility and communication impairments. These presentations and demonstrations were intended to help participants engage with the subject matter and were arranged by the organizing committee. After these presentations, the long list of priorities (n=25) generated by the survey were graphically presented to the group and placed as individual pieces of paper, in no particular order, on each table. The short-listing process consisted of each table reducing their list from 25 to 15 priorities through consensus discussions, which they subsequently presented and justified to the whole group for broader discussion.

A final selection of 10 priorities was then agreed through discussion by the whole group with a consensus on the inclusion of each priority reached by voting (raising a colored card for yes and no).

**Figure 1:**
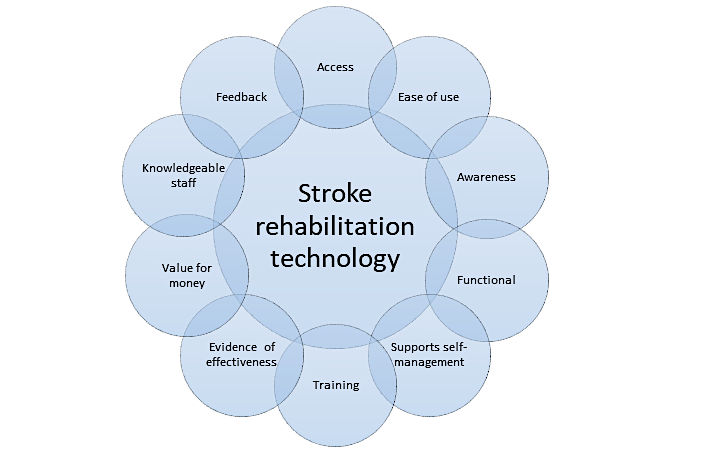
Top ten priority circle.

The following priorities were agreed at the end of the workshop. A short description is appended to each priority because the group felt that these clarifications were important to avoid ambiguity. They were initially ranked (based on the group vote) in the order set below. However, the workshop delegates requested the list should not be ranked, as the level of priority may differ according to the context and role of the individual, but they were happy that these were the 10 most important priorities. To encapsulate this lack of hierarchy, the final list was expressed as a circle (Figure 1).

The priorities for rehabilitation technology were as follows:

1. *Access to equipment:* This referred to users being able to access specific pieces of equipment without too much trouble and being able to use them within NHS facilities. The latter was particularly relevant to healthcare professionals using software apps that were blocked by NHS IT systems.
2. *Ease of use:* Although considered largely self-explanatory, there was a specific desire for devices to be operable with one hand and for all devices to be easy to use by all end users (ie, healthcare staff, stroke survivors, and their caregivers).
3. *Awareness:* This referred to the awareness of what technologies were actually available to the users in their local area as well as how they could access them.
4. *Functional:* Workshop delegates felt that any technology should be clearly focused on improving functional outcomes (ie, those that enhance activities of daily living whether related to mobility, speech, or cognition or memory).
5. *Supported self-management:* This was a priority identified as overlapping with other priorities (eg, access, ease of use, etc), but the consensus was that it should have its own position on the list. Technologies should, therefore, be designed with the ambition that they can be used to assist the user to manage their own condition by enabling them to practice rehabilitation activities.
6. *Training:* For all end users, training should available in accessible formats.
7. *Evidence of effectiveness:* This was widely debated as it was felt that definitive proof is unlikely to be achieved for technologies in the near future. The group felt that while a lack of research evidence on efficacy should not pose a barrier to a technology being adopted, the stakeholder community (users, policymakers, and technology developers) should work together to provide this evidence. Initially, this may be collated experiential evidence but should progress toward definitive evidence suitable for inclusion in practice guidelines.
8. *Value for money:* This term was originally described as “cost” but was altered so that the benefit of the technology, at both individual and societal levels, was considered relative to its monetary cost.
9. *Knowledgeable staff:* Stroke survivor end users felt that a technology was more likely to be used and be effective if their healthcare professional was knowledgeable (practically and theoretically) in its use.
10. *Feedback:* Where possible, technologies should provide information on general rehabilitation progress to users (therapists and patients) as well as detailed information on the performance of the specific activity. It was recognized that this was not always possible, for example, when using resistance bands. This information should be presented in an accessible format that takes into consideration the potential visual, cognitive, and communication impairments that people with stroke may be dealing with and should be available to healthcare professionals, provided this was agreed.

### Stakeholder Workshop 2: Generating New Ideas to Promote Rehabilitation Technology in Stroke

The second workshop took place in a neighboring city to broaden the stakeholder representation. Delegates (n=60) were recruited in the same manner as Workshop 1, with the organizing committee again deciding on the final delegate list to ensure an even distribution across the three stakeholder groups (users, technology developers, and policymakers). It is worth noting that 40 of the delegates attended Workshop 1. This was a deliberate decision to maintain some consistency. The aim of this workshop was to develop practical ideas for improving technology adoption considering the outcomes from the first workshop. To facilitate discussion, innovative rehabilitation practices (both models and use of technologies) were presented by local (Scotland and United Kingdom) and international (Italy and the Netherlands) speakers. The structure of the workshop, including presentation topics and speakers, was agreed by the organizing committee.

Following these presentations, delegates were organized into 7 tables of approximately 8 individuals with a mix of backgrounds (as per Workshop 1) to discuss the following question: “What practical steps could be taken to progress the aim of improving adoption of stroke rehabilitation technologies?”

The discussions from each table were summarized by a facilitator and presented to the whole group for further discussion. This process continued until clear outcomes, with consensus from the whole group, emerged. An agreement was finally reached on three practical steps to promote greater adoption of technologies in stroke rehabilitation: (1) an annual exhibition of rehabilitation technologies for all stakeholders, (2) formation of a network consisting of users, technology developers, and policymakers with the ambition of creating a road map for rehabilitation technologies, and (3) development of a consumer rater website inspired by websites such as TripAdvisor with the objectives of enhancing awareness of rehabilitation technologies, providing clear access to available research findings on the efficacy of these technologies, and allowing consumers to rate technologies on key attributes such as ease of use, value for money, and provision of feedback.

## Discussion

### Principal Findings

By providing the means to increase engagement with rehabilitation, technology has been shown to improve outcomes after stroke [6]. Despite growing evidence of efficacy, the adoption of these technologies by users (rehabilitation professionals, patients, and caregivers) is suboptimal [13]. A more integrated approach to technology development is required to ensure that this valuable resource is fully exploited [16]. Our study aimed to identify user priorities for rehabilitation technology and user-centered solutions to enhance the everyday adoption of these technologies by users: therapists, patients, and caregivers.

The 10 priorities identified by users through our survey and consensus workshops were similar to those reported by Hughes et al [17] and the Cumberland Consensus Working Group [18]. In particular, ease of use, evidence of effectiveness, access, and value for money have all been reported previously using questionnaire methodologies. Focus groups of pediatric and adult hemiplegic participants further confirm these enabling factors, adding motivation as a therapy “enabler” [19]. This is consistent with the feedback priority expressed by our stakeholder group.

### Strengths and Limitations

The use of facilitated workshops to develop a consensus among stakeholders was the strength of our approach since it provided the opportunity for broad face-to-face discussions among individuals with real and often contrasting experiences of using rehabilitation technologies. This open discourse was deemed necessary to reveal the range of factors involved and has been used successfully in similar priority setting exercises [12]. Furthermore, our inclusion of a second workshop that incorporated new delegates both confirmed the outcomes from the first workshop and generated practical steps to improve technology adoption. This information can be used to assist the industry to overcome the poor adoption of rehabilitation technologies.

The limitations of our study are similar to other approaches that depend on engagement with users, namely that the users responding to the survey and attending the workshops may not be typical of the entire user group in that they are likely to have a pre-existing interest in the area. Furthermore, there may be some social desirability bias, particularly from arranging therapists and patients around the same table [18].

### Conclusion

A series of workshops and surveys focusing on the adoption of technologies in stroke rehabilitation identified 10 key priorities by users (access to equipment, ease of use, awareness, functional, supported self-management, training, evidence of effectiveness, value for money, knowledgeable staff, and feedback). To improve adoption, practical steps including organization of an annual rehabilitation technology exhibition, formation of a network consisting of users, technology developers, and policymakers, and development of a consumer rater website were recommended.

## Appendix

Multimedia Appendix 1 Survey questions for stroke survivors, carers, and therapists.

## Abbreviations

CHSS: Chest Heart and Stroke Scotland
NHS: National Health Service
PSP: Priority Setting Partnership
