# Supplementary material for: Adoption of Stroke Rehabilitation Technologies by the User Community: Qualitative Study
Source: JMIR Rehabil Assist Technol. 2018 Aug 17;5(2):e15. doi: 10.2196/rehab.9219 (PMC6119213; doi:10.2196/rehab.9219)
Supplement: Multimedia Appendix 1 [file rehab_v5i2e15_app1.pdf]

Survey questions for stroke survivors

Hello,

We are a group of therapists and academics from across Scotland. Recently we were awarded a grant from the Scottish Universities Insight Institute to look at how equipment is used during stroke rehabilitation. While there are great examples of different types of equipment being used around the country (for example IPads, electrical stimulators, brain training games) we believe that if we are to improve recovery from stroke then the way we use technology needs a re-think. We are planning two workshops with therapists, NHS managers and people living with stroke.

Unfortunately we only have a small number of places at the workshops but would like to have as much input from people who have experienced rehabilitation after a stroke as possible whether that was for speech, mobility or cognitive impairment. In particular we want to know what you think of how equipment/technology was used (or not) during your rehabilitation. We would therefore really appreciate your responses to the following questions (on both sides of the form).

Question 1:

Which part of the United Kingdom do you live in?

Question 2:

Do you currently use technology (any special equipment) to help with your rehabilitation exercises/activities? Or have you used technology in the past? This could be anything from an IPad or mobile phone to a robot or computer game (e.g. a Wii) or even a resistance band for muscle strengthening

Yes (but in the past)   Yes and still doNo

Question 3:

Can you list of some of the things you typically used?

Question 4:

If you did use technology to help you with the activities was this suggested by someone from the NHS or was it something you discovered by yourself?

Yourself Healthcare worker

Question 5:

If you think technology should be used more in stroke rehabilitation what do you think are the main priorities?

Simply right down a list of 5 things you think are important. This doesn't just mean new technologies but could also be the way current ones are being used.

Question 6

Finally can you tell us what piece of equipment you found to be the most beneficial for helping you recover after your stroke and which was the least useful (or least liked).

Most valuable piece of equipment was____

Least valuable piece of equipment was____

Thank you

Survey questions for rehabilitation professional

Dear stroke rehabilitation professional,

We are a group of clinicians, academics and stroke survivors from across Scotland who were recently awarded a small grant from the Scottish Universities Insight Institute to look at the use of technology in stroke rehabilitation. While there are great examples of technology being used around the country, we believe that if we are to deliver the intensity and volume of rehabilitation required to make a real impact on patient outcomes, the way we use technology

in practice needs a re-think. The award is to run a series of three workshops including experts from around the world as well as local (UK) therapists, NHS managers and stroke survivors. Our aim is to determine if and how technology could play a greater role in stroke rehabilitation.

Although places at the workshops will be limited we would like to hear from the whole community of clinicians, academics/researchers, stroke survivors and carers we are therefore gather some basic views from the community across the UK regarding technology in stroke rehabilitation and would really appreciate your responses to the following questions.

Question 1:

Which region of the UK do you work in?

Question 2

Do you work in a) the community or b) in a hospital setting?

Question 3:

What is your profession?

Occupational Therapist Physiotherapist Speech and Language Therapist Orthotist Podiatrist Other? (e.g. Art Therapist, please state below?)

____

Question 4:

Can you write 5 words/phrases to describe your approach to stroke rehabilitation?

E.g. Practical, evidence-based, hands-on, problem-solving, education, functionally aimed, impairment based, tone reducing, normal movement, common sense, technology delivered, patient centred, tailored, based around aides, based around assistants

Question 5:

Can you tell us what (if any) technologies you use during practice?

Rehabilitation Technology can be any bit of equipment you use to help deliver the actual therapy or improve patient function (not simply assist in administrative duties, such as record keeping). This could mean anything from a resistance band to an IPad with interactive activities or even a robot.

Question 6

What do you think of the current use of technology in stroke rehabilitation?

Not enough about right too much already

Question 7

If you think more use can be made of technology in stroke rehabilitation what you think are the priorities, simply write down a list of 5 things you think are important. This doesn't just mean new technologies but could be the way current ones are being used.

Question 8

Finally would you like to make any other comments on the topic of rehabilitation technology for stroke ? if so please make them here.
